# HIF-1 Alpha Overexpression Correlates with Poor Overall Survival and Disease-Free Survival in Gastric Cancer Patients Post-Gastrectomy

**DOI:** 10.1371/journal.pone.0090678

**Published:** 2014-03-10

**Authors:** Li Chen, Yan Shi, Jing Yuan, Yalin Han, Rui Qin, Qian Wu, Baoqing Jia, Bo Wei, Lixin Wei, Guanghai Dai, Shunchang Jiao

**Affiliations:** 1 Department of Comprehensive Treatment Oncology, Chinese People's Liberation Army General Hospital, Beijing, China; 2 Department of Pathology, Chinese People's Liberation Army General Hospital, Beijing, China; 3 Department of Surgical oncology, Chinese People's Liberation Army General Hospital, Beijing, China; 4 Department of General surgery, Chinese People's Liberation Army General Hospital, Beijing, China; 5 Department of Medical oncology, Chinese People's Liberation Army General Hospital, Beijing, China; University of Texas MD Anderson Cancer Center, United States of America

## Abstract

**Background:**

Overall, gastric cancer prognosis remains poor. Detailed characterization of molecular markers that govern gastric cancer pathogenesis is warranted to establish innovative therapeutic options. HIF-1α overexpression has been linked to poor gastric cancer prognosis. However, though researched for years, the prognostic role of HIF-1α in gastric cancer is still controversial. Hence, the objective of the present study was to analyze the prognostic values of HIF-1α, TGF-β, VEGF and pERK1/2 in gastric cancer patients following gastrectomy.

**Methods:**

This study included 446 patients with confirmed gastric cancer who underwent gastrectomy in a single Chinese Cancer Center between 2005 and 2006. Clinicopathologic features, as well as immunohistochemical analysis of TGF-β, HIF-1α, VEGF and pERK1/2 were determined. Long-term survival of these patients was analyzed using univariate and multivariate analyses.

**Results:**

HIF-1α overexpression was more frequent in patients with hepatic metastases (71.6% versus 43.0% in those without hepatic metastases, *P* = 0.000, χ^2^ = 23.086) and more frequent in patients with peritoneum cavity metastasis (62.3% versus 43.0% in those without such metastasis, *P* = 0.000, χ^2^ = 13.691). In univariate analysis, patients with HIF-1α overexpression had a shorter disease-free survival (DFS) and overall survival (OS) than patients with weak-expression (DFS: NA VS. 16.8 m, *P* = 0.000, χ^2^ = 74.937; OS: NA VS. 25.5 m, *P* = 0.000, χ^2^ = 90.594). Importantly, HIF-1α overexpression was a promising prognostic marker for poor survival by multivariate analysis (DFS: HR 2.766, 95%CI 2.136–2.583, *P* = 0.000; OS: HR 3.529, 95%CI 2.663–4.667, *P* = 0.000).

**Conclusions:**

HIF-1α overexpression could be considered a useful independent prognostic biomarker in gastric cancer after gastrectomy, and is correlated to both a poor overall survival and disease-free survival in these patients. HIF-1α expression can be used to stratify patients at higher risk for poor prognosis, and is potentially an important therapeutic target in gastric cancer patients.

## Introduction

Gastric cancer (GC) is one of the most common malignancies in the world. Due to lack of specific early symptoms or effective tumor biomarkers, most patients with GC are not diagnosed until advanced stages. Although there has been great improvement in traditional treatments, the prognosis is still poor, and 30% to 50% of patients show relapse within 5 years of surgery and adjuvant chemotherapy [1], [2]. Thus, it is critical to identify specific markers and develop novel therapeutic strategies for advanced and recurrent gastric cancer.

Angiogenesis is an important determinant of tumor progression. Local tumor recurrence and distal metastasis are both dependent on neovascularization, which is regulated through angiogenesis factors. Several of these factors have been found to play an important role in regulating tumor angiogenesis, and are up regulated concomitantly with rapid growth and early metastasis [3]. Perhaps the best characterized markers are vascular endothelial growth factor (VEGF), hypoxia inducible factor-1 alpha (HIF-1α), extracellular signal-regulated kinases (ERK) and transforming growth factor- beta (TGF-β) [4].

Hypoxia and oxygen radicals co-operatively promote tumor angiogenesis [5] and cause the activation of HIF-1α, which in turn stimulates VEGF expression [6], [7]. TGF-β is also a major factor responsible for increased VEGF secretion. ERK is a downstream effector of the VEGF signaling pathway, which is regulated through angiogenesis. Clearly, these markers are intertwined as molecular components of angiogenesis. We hypothesized that these pathways might be responsible for tumor progression and metastasis in advanced gastric cancer.

In this study, the correlations of TGF-β, HIF-1α, VEGF and pERK1/2 expressions with clinicopathologic parameters and prognosis were evaluated in patients with gastric cancer. Furthermore, the influence of these markers on the recurrence and distant metastasis were assessed. The findings from the current study will contribute to predicting the risk of recurrence and metastasis of gastric cancer after gastrectomy, and help guide individualized treatment and development of new therapeutic targets.

## Materials and Methods

### Ethics statement

Signed informed consent was obtained from all study participants and all clinical investigations were conducted according to the principles outlined in the Declaration of Helsinki. Study protocols were approved by the Institutional Review Board of Chinese People's Liberation Army (PLA) General Hospital. All samples were procured from the tissue bank of Department of Pathology of PLA General Hospital.

### Patient selection and study design

A total of 446 patients with gastric cancer who underwent gastrectomy were enrolled in this study between January 2005 and December 2006 at the Chinese PLA General Hospital (China, Beijing). All patients had undergone initial curative gastrectomy. None of the patients received chemotherapy or radiotherapy before surgery. Only patients who had adequate paraffin embedded tumor specimens were included, and patients with adenosquamous carcinoma or neuroendocrine carcinoma were excluded. Patients lost during follow up or who died within one year of surgery were excluded from the analysis. Tumor staging was done according to the seventh edition of the American Joint Committee on Cancer/Union International Control Center TNM staging manual. Lesions staged as I to III with no evidence of metastatic disease were included.

Of the patients enrolled in this study, 348 (78.0%) were male and 98 (22.0%) were female, with a median age of 59.9 years (range 22.9–82.4 years). The median follow-up time was 63.9 months (range 55.0–78.8 months) until the end of the follow-up period (August 1, 2011). The clinicopathological features of the patients that were examined including gender, age, borrmann type, tumor size, tumor histological morphology, lauren classification, tumor differentiation (according to the WHO classification for gastric cancer in 2000), T category, N category, TNM stage (TNM 7th edition by American Joint Committee on Cancer), vascular invasion, perineural invasion, operation, and adjuvant chemotherapy. As of the follow-up end date, 19.7% of the patients (88/446) had hepatic metastases and 29.1% (130/446) had peritoneum cavity metastasis. The clinicopathological characteristics of patients are summarized in **Table 1**.

**Table 1:** Patient characteristics and Univariate analysis (n = 446).

Characteristics	N (%)	DFS			OS		
		months	P*	χ^2^	months	P*	χ^2^
**Age(median,years)**	59.9						
<60	224 (50.2)	37.9	0.044	4.042	51.0	0.015	5.860
≥60	222 (49.8)	23.5			30.9		
**Gender**							
Male	348 (78.0)	28.4	0.656	0.198	42.5	0.808	0.059
Female	98 (22.0)	25.3			38.9		
**Borrmann type**							
I	63 (14.1)	42.6	0.000	38.858	60.7	0.000	49.961
II+III	354 (79.4)	30.9			44.8		
IV	25 (5.6)	6.0			9.8		
V	4 (0.9)	5.8			4.0		
**Tumor Size**							
<5 cm	193 (44.3)	45.6	0.000	17.518	60.7	0.000	13.375
≥5 cm	253 (56.7)	19.6			30.6		
**Tumor Histological Morphology**							
Adenocarcinoma	287 (64.3)	45.7	0.000	28.041	60.7	0.000	31.692
Absolute signet ring cell carcinoma	71 (15.9)	15.1			21.3		
Mixed carcinoma	88 (19.8)	19.5			28.7		
**Lauren type**							
Intestinal	205 (46.0)	47.0	0.000	24.151	69.6	0.000	26.506
Diffuse	204 (45.7)	17.5			26.7		
Mixed type	37 (8.3)	47.7			63.9		
**Tumor differentiation** a							
Poor	354 (79.4)	24.7	0.033	4.569	33.0	0.006	7.702
Moderate and High	92 (20.6)	44.2			NA		
**Vessel invasion**							
No	279 (62.6)	33.9	0.021	5.289	48.5	0.013	6.223
Yes	167 (37.4)	18.4			29.5		
**Perineural invasion**							
No	313 (70.2)	37.9	0.004	8.196	51.0	0.005	7.967
Yes	133 (29.8)	18.9			27.6		
**T category**							
T1	17 (3.8)	NA	0.000	60.358	NA	0.000	57.479
T2	32 (7.2)	NA			NA		
T3	66 (14.8)	NA			NA		
T4	331 (74.2)	18.9			29.2		
**N category**							
N0	97 (21.7)	NA	0.000	116.151	NA	0.000	104.945
N1	94 (21.1)	47.2			69.6		
N2	109 (24.5)	22.6			29.5		
N3	146 (32.7)	11.5			20.5		
**TNM stage^b^**							
IA+IB	27 (6.1)	NA	0.000	163.206	NA	0.000	148.082
IIA	22 (4.9)	NA			NA		
IIB	83 (18.6)	NA			NA		
IIIA	83 (18.6)	32.1			46.7		
IIIB	106(23.8)	17.5			27.1		
IIIC	125(28.0)	10.2			17.8		
**Operation**							
D1	270(60.5)	24.0	0.435	0.610	32.8	0.170	1.883
D2	176(39.5)	33.9			50.4		
**Adjuvant chemotherapy**							
Yes	282(63.2)	45.7	0.000	65.261	63.9	0.000	41.181
No	164(36.8)	14.6			23.9		
**Hepatic metastases**							
Yes	88(19.7)	12.0	0.000	83.481	24.6	0.000	60.630
No	358(80.3)	46.2			60.7		
**Peritoneum cavity metastasis**							
Yes	130(29.1)	9.7	0.000	220.748	15.7	0.000	227.078
No	316(70.9)	55.4			NA		
**TGF-β**							
Weak-expression	265(59.4)	36.4	0.053	3.759	45.3	0.139	2.187
Over-expression	181(40.6)	26.1			36.6		
**HIF-1α**							
Weak-expression	229(51.3)	NA	0.000	74.937	NA	0.000	90.594
Over-expression	217(48.7)	16.8			25.5		
**VEGF**							
Weak-expression	252(56.5)	33.9	0.161	1.968	46.7	0.217	1.526
Over-expression	194(43.5)	24.0			34.8		
**pERK1/2**							
Weak-expression	297(66.6)	37.7	0.107	2.595	49.1	0.018	5.594
Over-expression	149(33.4)	19.8			27.8		

a Tumor differentiation according to the WHO classification for gastric cancer in 2000; ^b^TNM stage according to TNM 7th edition by AJCC(American Joint Committee on Cancer); NA Not arrival; *P<0.05.

In the absence of symptoms, physical examination was performed every 3–6 months for 5 consecutive years. Follow-up assessments consisted of physical examination, a complete blood count, liver function test, pulmonary, abdominal, and pelvic CT scan. The date of the first relapse and the date of death were recorded, and survival was calculated from the time of surgery until the last follow-up or death from any cause. Disease-free survival (DFS) was determined as the period between the date of surgery and the relapse diagnosis obtained by tests. Overall survival (OS) was defined as the interval in months measured between the date of resection and death for any cause.

### Tissue Microarray (TMA) Construction

For TMA construction, formalin-fixed, paraffin-embedded samples containing primary tumors and paired normal mucosa were retrieved from the archives of the Department of Pathology of our hospital. Representative areas of tissue were established by microscopic review of H&E stained slides, and 1.0 mm diameter cores were punched from the paraffin blocks. Three cores from primary cancer and one core from normal tissues (at least 2 cm distal to the tumor) were arrayed. TMAs were created using a Tissue Microarrayer (ALPHELYS, Minicore Tissue Arrayer Central Unit, France). All specimens were examined by at least two pathologists to prevent bias. Tumor and normal mucosa morphology on the arrays were validated as having high accordance with that of the whole archived section.

### Immunohistochemistry Staining

TGF-β, HIF-1α, VEGF and pERK1/2 expression were detected on the TMAs following citrate buffer (pH 6.0) antigen retrieval using standard methodology. Samples were incubated with primary antibody against TGF-β (Rabbit polyclonal Antibody, 1∶150, Abcam), HIF-1α (Rabbit Monoclonal Antibody, 1∶600, Epitomics), VEGF (Rabbit polyclonal, 1∶150, Abcam) or pERK1/2 (Rabbit Monoclonal Antibody, 1∶200, Cell Signaling), and then incubated with the second antibody (Dako REALTM EnVison TM Detection Syetem, Denmark). Tissue sections were counterstained with Mayer's hematoxylin. The positive controls were samples from our pathology specimen bank, while negative controls were experimental samples incubated with phosphate-buffered saline (PBS) instead of primary antibody.

### Immunohistochemical assessment

Immunohistochemical staining was evaluated independently by two pathologists without the knowledge of patient outcomes (double-blinded) according to the staining area and intensity [8]–[10]; the interobserver concordance was > 90%. In order to obtain accurate views of the tumors, three cores of representative regions were collected from primary cancer for tissue microarray. Pathologists comprehensively evaluated immunohistochemical staining of three cores, then provided a final score reflecting both the percentage of positive cells and the intensity of signal in positive cells (H-score range 0–12). Immunohistochemical score was applied as shown in Table 2, with the median H-score used as the cutoff. According to the H-scores of TGF-β, HIF-1α, VEGF and pERK1/2, each patient was assigned to either the overexpression group or the weak-expression group.

**Table 2:** Two procedures for evaluation of HER-2 expression.

IRS (Immunoreactive Score) modified by pathologists *			
Intensity of reaction	Points	Percentage of positive cells	Points
No reaction	0	No positive cells	0
Weak colour reaction	1	<25% positive cells	1
Moderate intensity	2	25–50% positive cells	2
Intense reaction	3	51–75% positive cells	3
		>75% positive cells	4

* IRS score (Immunoreactive Score) according to Remmele *et al* and Halon *et al* [8], [10].

### Statistical analysis

For statistical analysis, Statistical Package for Social Science (SPSS), version 19.0 was used. Correlations between the expressions of TGF-β, HIF-1α, VEGF and pERK1/2 were explored using Spearman's rank test, Correlations between clinicopathological factors and expression of TGF-β, HIF-1α, VEGF and pERK1/2 were examined using Pearson's Chi-Square test or Fisher's Exact test. The survival rate was calculated using the Kaplan–Meier method, and univariate survival analysis was performed using log-rank test. Multivariable analysis of prognostic factors was conducted by Cox proportional hazards model; *P*<0.05 was considered statistically significant.

## Results

### TGF-β, HIF-1α, VEGF and pERK 1/2 expressions in gastric cancer patients

**Figure 1:**
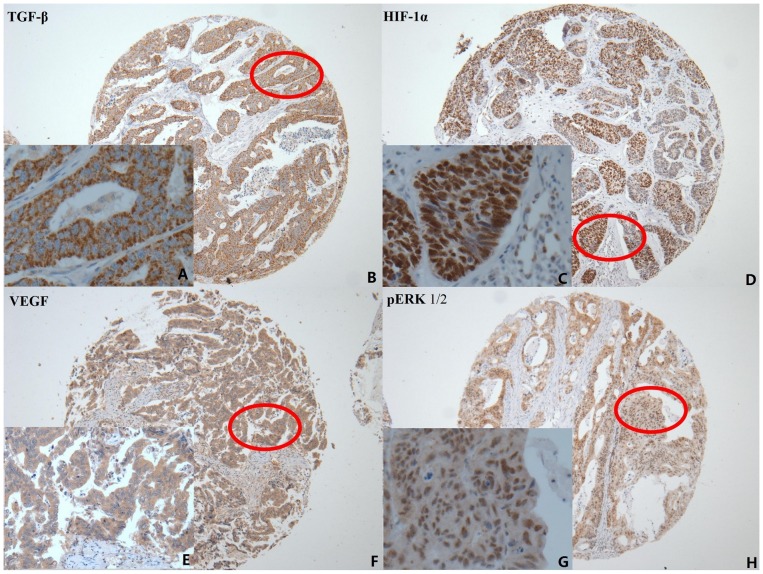
Immunohistochemical staining of TGF-β, HIF-1α, VEGF and pERK 1/2 expression in gastric cancer. A. Immunohistochemical staining of TGF-β was located mainly in the cytoplasm of tumor cells (positive expression ×400); B. TGF-β original magnification ×100; C. HIF-1α was located mainly in the nucleus of tumor cells (positive expression ×400); D. HIF-1α original magnification ×100; E. VEGF was located mainly in the cytoplasm of tumor cells (positive expression ×400); F. VEGF original magnification×100; G. pERK1/2 was located in the cytoplasm and nucleus of tumor cells (positive expression ×400); H. pERK1/2 original magnification ×100.

TGF-β (**Fig. 1A and 1B**) and VEGF (**Fig. 1E and 1F**) was dispersed granularly within the cytoplasm of tumor cells, expressed at varying levels (indicated by level intensity of color development). HIF-1α was expressed in tumor cell nuclei (**Fig. 1C and 1D**). pERK1/2 was observed both in tumor cell cytoplasm and nuclei (Fig. 1G and 1H). Among the total of 446 gastric cancer specimens, TGF-β overexpression was detected in 181 (40.6%), HIF-1α overexpression in 217 (48.7%), VEGF overexpression in 194 (43.5%), and pERK overexpression in 149 (34.3%).

Using Spearman's rank test, correlations between the expressions of TGF-β, HIF-1α, VEGF and pERK1/2 were explored. There is significant correlation between the overexpression of any two of these four proteins (*P*<0.05).

### Correlations between TGF-β, HIF-1α, VEGF and pERK1/2 expressions and clinicopathological factors

Correlations between clinicopathological factors and expression of TGF-β, HIF-1α, VEGF and pERK1/2 were observed using Pearson's Chi-Square test or Fisher's Exact test. The detailed characteristics are shown in Table 3 and Table 4. TGF-β overexpression was more frequent in patients with peritoneum cavity metastasis (50.8% versus 36.4% in those without peritoneum cavity metastasis, *P* = 0.005, χ^2^ = 7.895). HIF-1α overexpression was more frequent in patients with hepatic metastases (71.6% versus 43.0% in those without hepatic metastases, *P* = 0.000, χ^2^ = 23.086) and was more frequent in patients with peritoneum cavity metastasis (62.3% versus 43.0% in those without peritoneum cavity metastasis, *P* = 0.000, χ^2^ = 13.691).

**Table 3:** TGF-β and HIF-1α expressions and Clinicopathologic characteristics.

Characteristics	TGF-β			HIF-1α		
	Low	High	*P* a	Low	High	*P* a
	265(59.4)	181(40.6)		229(51.3)	217(48.7)	
**Age**						
<60	135(60.3)	89(39.7)	0.713	124(55.4)	100(44.6)	0.089
≥60	130(58.6)	92(41.4)		105(47.3)	117(52.7)	
**Gender**						
Male	204(58.6)	144(41.4)	0.519	176(50.6)	172(49.4)	0.540
Female	61(62.2)	37(37.8)		53(54.1)	45(45.9)	
**Borrmann type**						
I	40(63.5)	23(36.5)	0.183	28(44.0)	35(55.6)	0.435
II+III	207(58.5)	147(41.5)		186(52.5)	168(47.5)	
IV	14(56.0)	11(44.0)		14(56.0)	11(44.0)	
V	4 (100.0)	0 (0.0)		1(25.0)	3 (75.0)	
**Tumor Size**						
<5 cm	125(64.8)	68(35.2)	0.044	98(50.8)	95(49.2)	0.834
≥5 cm	140(55.3)	113(44.7)		131(51.8)	122(48.2)	
**Tumor Histological Morphology**						
Adenocarcinoma	175(61.0)	112(39.0)	0.644	156(54.4)	131(45.6)	0.230
Absolute signet ring cell carcinoma	41(57.7)	30(42.3)		33(46.5)	38(53.5)	
Mixed carcinoma	49(55.7)	39(44.3)		40(45.5)	48(54.5)	
**Lauren type**						
Intestinal	125(61.0)	80(39.0)	0.366	107(52.2)	98(47.8)	0.854
Diffuse	115(56.4)	89(43.6)		102(50.0)	102(50.0)	
mixed type	25(67.6)	12(32.4)		20(54.1)	17(45.9)	
**Tumor differentiation**						
Poor	209(59.0)	145(41.0)	0.750	183(51.7)	171(48.3)	0.772
Moderate and High	56(60.9)	36(39.1)		46(50.0)	46(50.0)	
**Vessel invasion**						
Yes	160(57.3)	119(42.7)	0.250	142(50.9)	137(49.1)	0.806
No	105(62.9)	62(37.1)		87(52.1)	80(47.9)	
**Perineural invasion**						
Yes	181(57.8)	132(42.2)	0.294	166(53.0)	147(47.0)	0.273
No	84(63.2)	49(36.8)		63(47.4)	70(52.6)	
**T category**						
T1	12(70.6)	5(29.4)	0.630	8(47.1)	9(52.9)	0.112
T2	18(56.3)	14(43.8)		15(46.9)	17(53.1)	
T3	36(54.5)	30(45.5)		43(65.2)	23(34.8)	
T4	199(60.1)	132(39.9)		163(49.2)	168(50.8)	
**N category**						
N0	65(67.0)	32(33.0)	0.069	53(54.6)	44(45.4)	0.147
N1	47(50.0)	47(50.0)		46(48.9)	48(51.1)	
N2	61(56.0)	48(44.0)		47(43.1)	62(56.9)	
N3	92(63.0)	54(37.0)		83(56.8)	63(43.2)	
**TNM stage**						
IA+IB	20(74.1)	7(25.9)	0.005	15(55.6)	12(44.4)	0.775
IIA	15(68.2)	7(31.8)		13(59.1)	9(40.9)	
IIB	50(60.2)	33(39.8)		43(51.8)	40(48.2)	
IIIA	35(42.2)	48(57.8)		37(44.6)	46(55.4)	
IIIB	61(57.5)	45(42.5)		54(50.9)	52(49.1)	
IIIC	84(67.2)	41(32.8)		67(53.6)	58(46.4)	
**Hepatic metastases**						
No	219(61.2)	139(38.8)	0.128	204(57.0)	154(43.0)	0.000
Yes	46(52.3)	42(47.7)		25(28.4)	63(71.6)	
**Peritoneum cavity metastasis**						
No	201(63.6)	115(36.4)	0.005	180(57.0)	136(43.0)	0.000
Yes	64(49.2)	66(50.8)		49(37.7)	81(62.3)	

a Pearson's Chi-Square test or Fisher's Exact test, P<0.05.

**Table 4:** VEGF and pERK expressions and Clinicopathologic characteristics.

Characteristics	VEGF			pERK		
	Low	High	*P* a	Low	High	*P* a
	252(56.5)	194(43.5)		297(65.7)	149(34.3)	
**Age**						
<60	120(53.6)	104(46.4)	0.210	147(65.6)	77(34.4)	0.664
≥60	132(59.5)	90(40.5)		150(67.6)	72(32.4)	
**Gender**						
Male	192(55.2)	156(44.8)	0.286	236(67.8)	112(32.2)	0.302
Female	60(61.2)	38(38.8)		61(62.2)	37(37.8)	
**Borrmann type**						
I	39(61.9)	24(38.1)	0.642	36(57.1)	27(42.9)	0.090
II+III	195(55.1)	159(44.9)		238(67.2)	116(32.8)	
IV	16(64.0)	9(36.0)		21(84.0)	4(16.0)	
V	2 (50.0)	2 (50.0)		2(50.0)	2 (50.0)	
**Tumor Size**						
<5 cm	113(58.5)	80(41.5)	0.446	135(69.9)	58(30.1)	0.189
≥5 cm	139(54.9)	114(45.1)		162(64.0)	91(36.0)	
**Tumor Histological Morphology**						
Adenocarcinoma	153(53.3)	134(46.7)	0.001	191(66.6)	96(33.4)	0.903
Absolute signet ring cell carcinoma	54(76.1)	17(23.9)		46(64.8)	25(35.2)	
Mixed carcinoma	45(51.1)	43(48.9)		60(68.2)	28(31.8)	
**Lauren type**						
Intestinal	109(53.2)	96(46.8)	0.400	134(65.4)	71(34.6)	0.822
Diffuse	122(59.8)	82(40.2)		137(67.2)	67(32.8)	
mixed type	21(56.8)	16(43.2)		26(70.3)	11(29.7)	
**Tumor differentiation**						
Poor	202(57.1)	152(42.9)	0.640	238(67.2)	116(32.8)	0.574
Moderate and High	50(54.3)	42(45.7)		59(64.1)	33(35.9)	
**Vessel invasion**						
Yes	167(59.9)	112(40.1)	0.065	187(67.0	92(33.0)	0.802
No	85(50.9)	82(49.1)		110(65.9)	57(34.1)	
**Perineural invasion**						
Yes	184(58.8)	129(41.2)	0.136	208(66.5)	105(33.5)	0.924
No	68(51.1)	65(48.9)		89(66.9)	44(33.1)	
**T category**						
T1	12(70.6)	5(29.4)	0.471	10(58.2)	7(41.2)	0.293
T2	20(62.5)	12(37.5)		17(53.1)	15(46.9)	
T3	34(51.5)	32(48.5)		43(65.2)	23(34.8)	
T4	186(56.2)	145(42.8)		227(68.6)	104(31.4)	
**N category**						
N0	64(66.0)	33(34.0)	0.091	66(68.0)	31(32.0)	0.619
N1	51(54.3)	43(45.7)		61(64.9)	33(35.1)	
N2	64(58.7)	45(41.3)		68(62.4)	41(37.6)	
N3	73(50.0)	73(50.0)		102(69.9)	44(30.1)	
**TNM stage**						
IA+IB	20(74.1)	7(25.9)	0.240	14(50.0)	13(50.0)	0.540
IIA	13(59.1)	9(40.9)		14(50.0)	8(50.0)	
IIB	50(60.2)	33(39.8)		56(67.8)	27(32.2)	
IIIA	50(60.2)	33(39.8)		59(69.4)	24(30.6)	
IIIB	55(51.9)	51(48.1)		68(65.3)	38(34.7)	
IIIC	64(41.2)	61(48.8)		86(67.2)	39(32.8)	
**Hepatic metastases**						
No	206(57.5)	152(42.5)	0.372	240(67.0)	118(33.0)	0.686
Yes	46(52.3)	42(47.7)		57(64.8)	31(35.2)	
**Peritoneum cavity metastasis**						
No	174(55.1)	142(44.9)	0.339	218(69.0)	98(31.0)	0.094
Yes	78(60.0)	58(40.0)		79(60.8)	51(39.2)	

a Pearson's Chi-Square test or Fisher's Exact test, P<0.05.

### Univariate analysis

Using the Kaplan-Meier method and the log-rank test, correlations between clinicopathological factors and patient outcomes were evaluated. Of the 446 patients, 295 (66.1%) developed recurrence and/or metastasis, and 263 (59.0%) died prior to the follow-up end date (August 1, 2011). Median DFS was 28.1 months and median OS was 40.2 months. The 3-year and 5-year overall survival rates were 52% and 39%, respectively.

**Figure 2:**
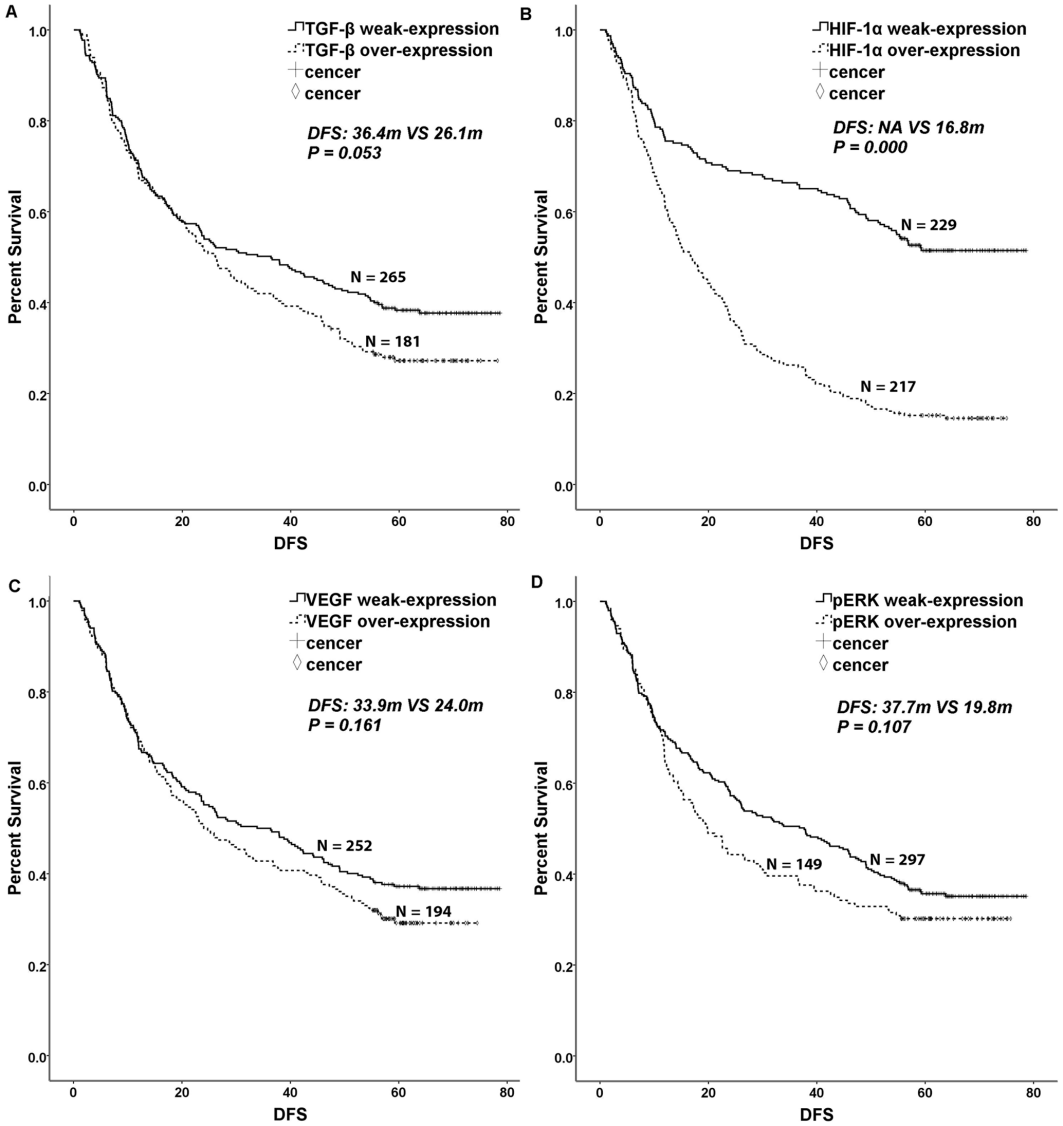
Kaplan-Meier curves for disease-free survival. TGF-β, HIF-1α, VEGF and pERK 1/2 overexpression were divided into an overexpression group and a weak-expression group. A log-rank test was used to calculate significance. A. Disease-free survival curves stratified by TGF-β expression (*P* = 0.053). B. Disease-free survival curves stratified by HIF-1α expression (*P* = 0.000). C. Disease-free survival curves stratified by VEGF expression (*P* = 0.161). D. Disease-free survival curves stratified by pERK 1/2 expression (*P* = 0.107).

**Figure 3:**
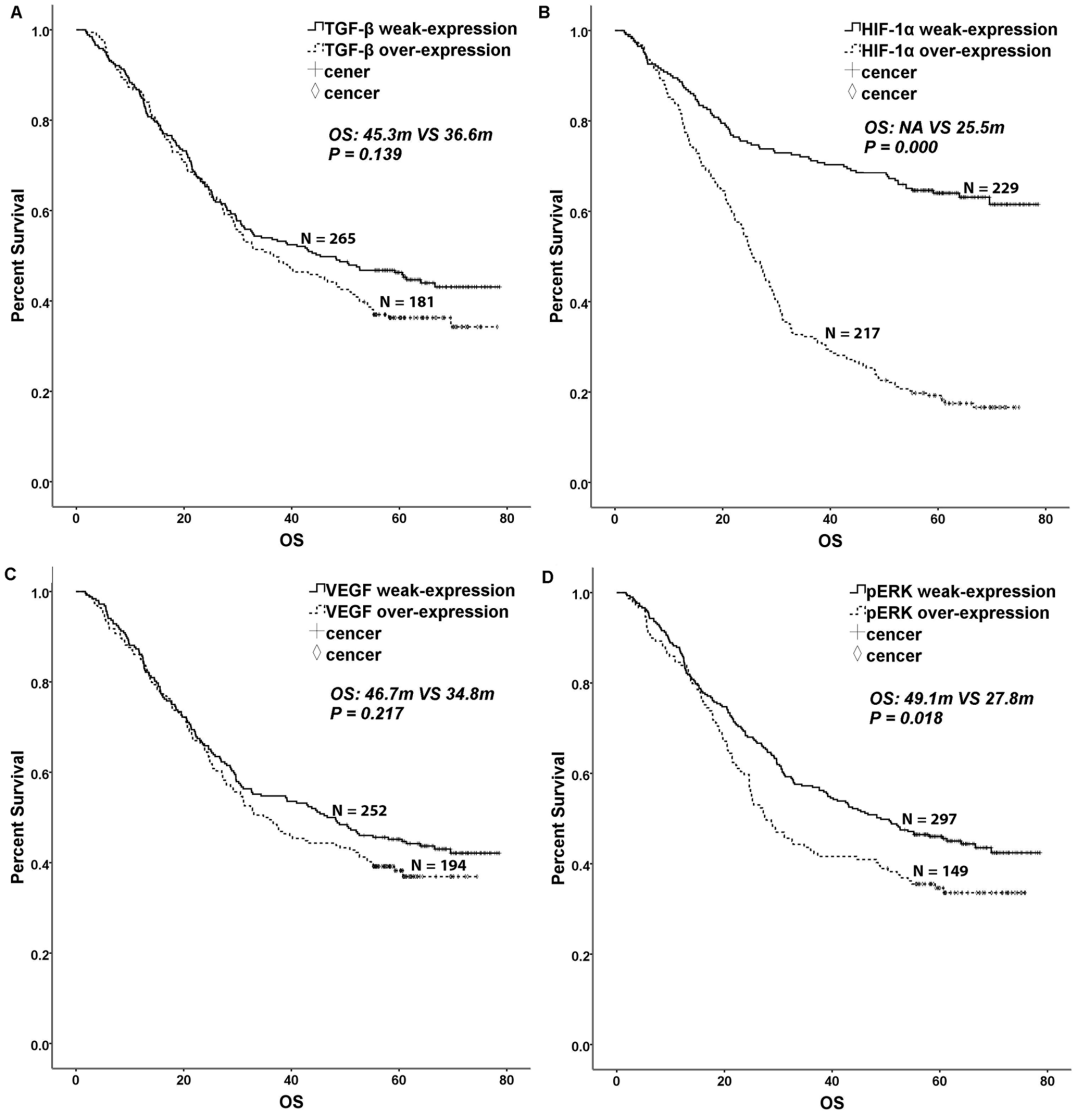
Kaplan-Meier curves for overall survival. TGF-β, HIF-1α, VEGF and pERK 1/2 overexpression were divided into an overexpression group and a weak-expression group. A log-rank test was used to calculate significance. A. Overall survival curves stratified by TGF-β expression (*P* = 0.139). B. Overall survival curves stratified by HIF-1α expression (*P* = 0.000). C. Overall survival curves stratified by VEGF expression (*P* = 0.217). D. Overall survival curves stratified by pERK 1/2 expression (*P* = 0.018).

The widely accepted prognostic factors of borrmann type, tumor size, tumor histology, lauren type, tumor differentiation, vessel invasion, perineural invasion, T category, N category, TNM stage and adjuvant chemotherapy were associated with DFS and OS in gastric cancer after gastrectomy. Hepatic metastases and peritoneum cavity metastasis were associated with OS in gastric cancer after gastrectomy. Patients displaying weak TGF-β expression had a longer DFS than those displaying overexpression of TGF-β, with a *P* value close to 0.05. However, no difference in OS was observed (DFS: 36.4 m VS. 26.1 m, *P* = 0.053, χ^2^ = 3.759, Tab 1, Fig. 2A; OS: 45.3 m VS. 36.6 m, *P* = 0.139, χ^2^ = 2.187, Tab 1, Fig. 3A). Patients with HIF-1α weak-expression had a longer survival time than those with HIF-1α over-expression (DFS: NA VS. 16.8 m, *P* = 0.000, χ^2^ = 74.937, Tab 1, Fig. 2B; OS: NA VS. 25.5 m, *P* = 0.000, χ^2^ = 90.594, Tab 1, Fig. 3B). Patients with pERK weak-expression had a longer OS than patients with over-expression of pERK (DFS: 37.7 m VS. 19.8 m, *P* = 0.107, χ^2^ = 2.595, Tab 1, Fig. 2D; OS: 49.1 m VS. 27.8 m, *P* = 0.018, χ^2^ = 5.594, Tab 1, Fig. 3D). However, VEGF expression was not correlated with DFS and OS (*P*>0.1, Tab 1, Fig. 2C, Fig. 3C).

### Multivariate analysis

Parameters with *P*-values of ≤0.1 in the univariate analysis were included in the multivariate analysis using the Cox proportional hazards. The results are summarized in Table 5. Results from the Cox proportional hazards model using the backward stepwise method indicated that HIF-1α overexpression was an independent prognostic factor in predicting DFS and OS. Patients with HIF-1α overexpression had a shorter survival and higher risk of recurrence and death than patients with HIF-1α weak-expression (DFS: HR 2.766, 95%CI 2.136–2.583, P = 0.000; OS: HR 3.529, 95%CI 2.663–4.667, P = 0.000, Table 5).

**Table 5:** Multivariate analysis of significant prognostic factors for survival in patients with gastric carcinoma.

Variables	DFS			OS		
	P *	HR	95%CI	P *	HR	95%CI
Age(<60years, ≥60years)	0.968	1.005	0.787–1.283	0.676	1.058	0.813–1.376
Borrmann type(I, II+III, IV, V)	0.000	1.671	1.306–2.138	0.000	1.950	1.475–2.578
Tumor Size(<5 cm, ≥5 cm)	0.311	1.138	0.886–1.463	0.706	1.052	0.808–1.369
Histological Morphology(A, S, M)1	0.481	1.036	0.939–1.143	0.505	1.305	0.935–1.146
Lauren type(I, D, M)2	0.259	1.109	0.927–1.327	0.226	1.124	0.930–1.360
Tumor differentiation(P/D, M/D+H/D)3	0.894	0.978	0.702–1.362	0.437	0.866	0.602–1.245
Vessel invasion(YES, NO)	0.239	1.157	0.908–1.475	0.152	1.201	0.935–1.544
Perineural invasion(YES, NO)	0.661	1.060	0.816–1.377	0.871	1.016	0.836–1.235
T category(T1, T2, T3, T4)	0.444	1.103	0.858–1.420	0.723	1.051	0.798–1.384
N category(N0, N1, N2, N3)	0.219	1.382	0.825–2.313	0.314	1.336	0.760–2.347
TNM stage(I, II, IIIA, IIIB, IIIC)	0.000	2.008	1.781–2.265	0.000	1.654	1.522–1.798
Adjuvant chemotherapy(YES, NO)	0.000	0.346	0.268–0.447	0.000	0.330	0.252–0.432
TGF-β expression(W, O)4	0.143	1.191	0.943–1.506	0.319	1.136	0.884–1.460
HIF-1α expression(W, O)4	0.000	2.766	2.136–2.583	0.000	3.529	2.663–4.667
pERK1/2 expression(W, O)4	0.084	1.249	0.971–1.606	0.009	1.420	1.092–1.845

DFS, Disease-free survival; OS, Overall Survival; HR, hazard ratio; CI, Confidence interval; *P<0.05; 1, A, Adenocarcinoma, S, Absolute signet ring cell carcinoma, M, Mixed carcinoma; 2, I, Intestinal, D, Diffuse, M, Mixed type; 3, P/D, Poor differentiation, M/D, Moderate differentiation, H/D High differentiation; 4, W, Weak-expression, O, Overexpression.

## Discussion

Metastasis remains a major cause of treatment failure for patients with cancer, and angiogenesis is for metastasis to occur. In 1970s, Folkman found that tumor growth and metastasis are dependent on angiogenesis when the tumor size exceeds 2–3 mm [7]. Factors that can be used to predict the metastatic potential of cancer have been actively sought for several decades. The most significant finding from the current study is that TGF-β, HIF-1α, VEGF and pERK, all proangiogenic and angiogenic factors found within solid tumors and up regulated in malignancy, are linked to poor prognosis with disease progression [11]–[12], [4].

Hypoxia is one of the most important environmental factors that induce cancer metastasis [13]–[17]. Each step of the metastatic process, from the initial epithelial-mesenchymal transition (EMT) to the ultimate organotropic colonization, can potentially be regulated by hypoxia, suggesting a master regulator role for hypoxia and HIFs in metastasis. Furthermore, modulation of cancer stem cell self-renewal by HIFs may also contribute to the hypoxia-regulated metastasis program [15]. HIF-1α regulates both transcription factors and chromatin modifiers to induce metastasis in an EMT-dependent or -independent manner. In addition, various targets regulated by HIF-1α that mediate other biological effects such as metabolism might also contribute to metastasis [16]. HIF-1α expression is correlated with poor prognostic clinicopathologic characteristics and survival in different cancers [18]. In an analysis of pancreatic adenocarcinoma, Wei *et al* found that hypoxia significantly promotes cell proliferation and migration, resulting in metastasis both *in vitro* and *in vivo* [17]. Wang *et al* examined the possible role for HIF-1α and HIF-2α in the process of invasiveness and metastasis of gastric cancer during hypoxia, with involvement of the JNK signal pathway. Their results showed that HIF-1α and HIF-2α were more highly expressed in metastatic gastric cancers compared to non-metastatic carcinomas [19], indicating that HIF-1α is likely a major determinant of invasion and metastasis in several tumor types.

In fact, the targeted inhibition of HIF-1α has been shown to inhibit the growth of gastric tumors in animals [20], [21]. Furthermore, the prognostic role of HIF-1α in gastric tumor had been searched in many trials. However, though researched for years, the prognostic role of hypoxia-inducible factor 1 alpha (HIF-1α) in gastric cancer is still controversial. In a meta-analysis performed by Zhang et al. [22], involving 12 trials (1,555 patients), it was reported that HIF-1α expression was significantly correlated with poor overall survival of gastric cancer patients (HR = 1.34, 95%CI: 1.13–1.58; *P* = 0.0009), but not with poor disease free survival of gastric cancer patients (HR = 1.67, 95%CI: 0.99–2.82; *P* = 0.06). This is also the point where the novelty of our current manuscript becomes apparent. Of the 12 studies that formed the basis of the aforementioned meta-analysis, the largest sample size was 216 [22]. The sample size in our study was 446 patients. Hence, the current study is the single largest sample size in which correlation of HIF-1α and prognosis of gastric cancer was evaluated. Our univariate analysis revealed that patients with HIF-1α overexpression had both a shorter disease-free survival (DFS) and overall survival (OS) than patients with weak-expression. Importantly, HIF-1α overexpression was also a promising prognostic marker for poor survival by multivariate. This is in stark contrast to the conclusion of the aforementioned meta-analysis [22], where it was not related to DFS. Hence, our study shows for the first time that HIF-1α overexpression is correlated to not only OS, but also DFS, in gastric cancer patients. Through rational extrapolation such a finding will come into the equation when novel therapeutics targeting HIF-1α, will be evaluated.

In the present study, increased overexpression of HIF-1α was observed in GC patients with peritoneum cavity metastasis. These results are consistent with previous basic research studies. Using *in vivo* metastatic models, Miyake *et al* provided a possible mechanism in which peritoneal dissemination of gastric cancer develops via a vascular network, whereby HIF-1α activates tumor angiogenesis [23]. Matsuo *et al* showed that HIF-1α expression was significantly associated with the high incidence of hepatic metastasis in pancreatic ductal adenocarcinoma [24]. Shimomura *et al* analyzed patients who underwent curative resection and found that overexpression of HIF-1α was an independent risk factor in colorectal liver metastasis [25]. In work presented here, increased overexpression of HIF-1α was observed in GC patients with hepatic metastases, a result consistent with the above studies showing a close link between HIF-1α and liver metastasis.

Many studies indicate that TGF-β signaling can act as either a tumor promoter or a tumor suppressor. Some investigators have explored the role of TGF-β1 in lung cancer, finding in patients that TGF-β predicted poor distant metastasis-free survival (DMFS) and poor brain metastasis after adjustment for other factors. They also found in culture that transfection with TGF-β stimulated migration and invasion of lung cancer cells, suggesting that TGF-β may be involved in increased metastatic potential [26], [27], [14]. In addition, cancer cells over-expressing active TGF-β increased metastatic ability, and targeting of TGF-signaling prevented metastasis in several cancers such as breast and prostate [28]–[30]. Others have suggested that TGF-β protein levels might independently predict survival in patients with lung adenocarcinoma [27], [31]. In those studies, TGF-β expression in primary lung cancer tissues was higher among patients with pulmonary metastases than in patients without such metastases. Additional work has investigated differences in TGF-β levels and their association with colorectal cancer (CRC) progression, finding that TGF-β levels in this context are a robust predictor of disease relapse [32], [33]. In gastric cancer, Comerci *et al* found that secreted TGF-β1 might indirectly promote tumor progression [34]. Ottaviano *et al* showed that TGF-β1-mediated crosstalk between gastric cancer cells and stromal elements influenced cell surface- and pericellular matrix-degrading potential *in vitro* [35]. Fu *et al* reported that TGF- significantly promoted the invasion and metastasis of the gastric cancer cell lines SGC7901 and BGC823 by increasing fascin1 expression via the ERK and JNK signaling pathways [36]. Additionally, Ma *et al* concluded that the secretion of TGF-β by both tumor and stromal cells might play important roles in development and maintenance of the tumor microenvironment [37]. Researchers have also examined human tissues with early gastric cancer (EGC) and advanced gastric cancer (AGC). Positive staining for the intracellular form of TGF-β was found in 59.1% of EGC, and 66.7% of AGC samples. In contrast, there was no difference in the expression of TGF-β in relation to Helicobacter pylori (*Hp*) infection, Lauren's classification or lymph node involvement. Moreover, clinical studies showed the positive correlation of TGF-β expression with lymph node metastasis and poor prognosis in gastric carcinoma [38], [39]. Similar to these results we have found in the current study that TGF-β overexpression was more frequent in patients with peritoneum cavity metastasis than in patients without such metastasis. Patients with TGF-β overexpression had a shorter disease-free survival time than those with TGF-β weak-expression in the univariate analysis, while it was excluded from the multivariate analysis. Therefore, our findings indicate that TGF-β might facilitate cancer metastasis but does not constitute an independent factor.

It should be noted that one limitation of this study is that the data used was limited and retrospective. Further research will be important to better understand the relationship between the above markers and survival.

### Conclusions

Our work here suggests that overexpression of HIF-1α could be an important indicator of poor prognosis in gastric cancer after gastrectomy. Although further work will be needed to validate these conclusions in a clinical setting, HIF-1α overexpression correlated well with hepatic metastases and peritoneum cavity metastasis in patients with GC. In addition, further research into the relationship between antiangiogenic therapy and metastasis of gastric cancer may provide additional potential drug targets, resulting in therapies that can enhance the clinical benefits of antiangiogenic treatment.
